# Implementation of health-related quality of life in the German TraumaRegister DGU® – first results of a pilot study

**DOI:** 10.1186/s12955-024-02261-y

**Published:** 2024-06-05

**Authors:** Carina Jaekel, Ulrike Nienaber, Anne Neubert, Oliver Kamp, Lisa Wienhöfer, Andre Nohl, Marc Maegele, Helena Duesing, Christoph J. Erichsen, Stephan Frenzel, Rolf Lefering, Sascha Flohe, Dan Bieler, Outcome Study Group of the Committee on Emergency Medicine, Intensive Care, Trauma Management (Sektion NIS) of the German Trauma Society (DGU), I. Gnass, S. M. Heining, S. Kaske, E. Kollig, U. Polak, S. Simmel, J. Sturm, S. Thelen, R. Volland

**Affiliations:** 1 Department of Orthopaedics and Traumatology, University Hospital and Medical Faculty, Heinrich-Heine-University Duesseldorf, Duesseldorf, Germany; 2 AUC-Academy for Trauma Surgery, Cologne, Germany; 3 Department of Trauma Surgery, Hand and Reconstructive Surgery, University Hospital Essen, Essen, Germany; 4 Department of Emergency Medicine, BG Klinikum Duisburg, Duisburg, 47249 Germany; 5 Cologne-Merheim Medical Center (CMMC), Department of Trauma and Orthopedic Surgery, University Witten/Herdecke, Campus Cologne-Merheim, Cologne, Germany; 6 Department of Trauma-, Hand- and Reconstructive Surgery, University Hospital Muenster, Muenster, Germany; 7 Department of Trauma Surgery, BG Trauma Center Murnau, Murnau Am Staffelsee, Murnau, 82418 Germany; 8 Department of Orthopedics and Trauma Surgery, Medical University of Vienna, Vienna, Austria; 9 Institute for Research in Operative Medicine (IFOM), Universität Witten/Herdecke, Ostmerheimer Str.200, Haus 38, Cologne, 51109 Germany; 10 Department of Trauma, Orthopaedics and Hand Surgery, Städt. Klinikum Solingen, Solingen, Germany; 11 Department of Orthopedics and Trauma Surgery, Reconstructive Surgery, Hand Surgery, Plastic Surgery and Burn Medicine, German Armed Forces Central Hospital, Koblenz, Germany

**Keywords:** Outcome, Health-related quality of life, Polytrauma, Trauma registry

## Abstract

**Background:**

Approximately 30,000 people are affected by severe injuries in Germany each year. Continuous progress in prehospital and hospital care has significantly reduced the mortality of polytrauma patients. With increasing survival rates, the functional outcome, health-related quality (hrQoL) of life and ability to work are now gaining importance. Aim of the study is, the presentation of the response behavior of seriously injured patients on the one hand and the examination of the factors influencing the quality of life and ability to work 12 months after major trauma on the other hand. Building on these initial results, a standard outcome tool shall be integrated in the established TraumaRegister DGU® in the future.

**Methods:**

In 2018, patients [Injury Severity Score (ISS) ≥ 16; age:18–75 years] underwent multicenter one-year posttraumatic follow-up in six study hospitals. In addition to assessing hrQoL by using the Short-Form Health Survey (SF-12), five additional questions (treatment satisfaction; ability to work; trauma-related medical treatment; relevant physical disability, hrQoL as compared with the prior to injury status) were applied.

**Results:**

Of the 1,162 patients contacted, 594 responded and were included in the analysis. The post-injury hrQoL does not show statistically significant differences between the sexes. Regarding age, however, the younger the patient at injury, the better the SF-12 physical sum score. Furthermore, the physically perceived quality of life decreases statistically significantly in relation to the severity of the trauma as measured by the ISS, whereas the mentally perceived quality of life shows no differences in terms of injury severity. A large proportion of severely injured patients were very satisfied (42.2%) or satisfied (39.9%) with the treatment outcome. It should be emphasized that patients with a high injury severity (ISS > 50) were on average more often very satisfied with the treatment outcome (46.7%). A total of 429 patients provided information on their ability to work 12 months post-injury. Here, 194 (45.2%) patients had a full employment, and 58 (13.5%) patients were had a restricted employment.

**Conclusion:**

The present results show the importance of a structured assessment of the postinjury hrQoL and the ability to work after polytrauma. Further studies on the detection of influenceable risk factors on hrQoL and ability to work in the intersectoral course of treatment should follow to enable the best possible outcome of polytrauma survivors.

## Introduction

Around 4.4 million people worldwide die due to injuries every year and are responsible for around 8% of all deaths and account for about 8% of all deaths [1]. Among people aged 5 to 29 years, three of the five leading causes of death are injury-related, namely traffic accidents, manslaughter and suicide [1]. In Germany, approximately 10 million people suffer an injury each year. Of these, about 30,000 are seriously injured in an accident [2, 3]. Furthermore, injuries and violence account for an estimated 10% of all years of life with disabilities. In contrast, the continued development of medical care in the prehospital and clinical settings has led to significant reductions in trauma-related mortality over the past four decades [4]. As survival rates increase, the health-related quality of life (hrQoL) of seriously injured patients becomes increasingly important. This is because, despite the reduction in mortality, trauma survivors often suffer from long-term impairments, severe disabilities, or increased morbidity [5–8]. Especially, physical, social as well as psychological consequences have been identified [5, 6, 8–10]. Long-term outcomes appear to be dependent on demographic factors (age, sex, and body mass index), injury severity, psychological components, socioeconomic status, and educational level [5, 11–13]. Hence, it is of crucial importance to assess the clinical and functional outcomes of survivors after severe trauma. In this regard, the investigation of hrQoL, particularly in the form of patient-reported outcome measures (PROMs), is coming to the forefront of research [14]. Previous work in the area of hrQoL after injury is scarce, either including only small study populations, being retrospective in design, or examining only a specific entity of injury [11, 15–19].

### TraumaRegister DGU®

With one of the largest serious injury registries worldwide, the TraumaRegister DGU® (TR-DGU), is documenting the prehospital and hospital data of trauma patients in a standardized and pseudonymized manner annually. In addition to quality assurance, the registry also serves scientific purposes and thus the identification of the above-mentioned risk factors for mortality and complications after sever trauma. Based on the well-established TR-DGU, we assume that with the implementation of the recording of hrQoL within the TR-DGU the documentation of large patient numbers would be possible. In the future, such a registry-based outcome assessment of trauma survivors might represent a valuable research tool as well as a tool for quality measurements.

### Objectives

The present study reports on the first experiences with the implementation of the multicentric collection of patient-reported and hrQoL measurements of patients 12 months post-injury. It attempts to derive first perceptions from pilot phase. The research questions arising from this are:

1. How many of the included trauma patients participate in the study and is the response behavior dependent on different factors (age, sex, injury severity, clinical course, outcome)?
2. What is the patient-reported and hrQoL in severely injured patients 12 months post-injury and which of the above listed factors, such as the distribution of injury types and sociodemographic factors, determine the hrQoL?
3. How many patients are able to return to work 12 months post-injury?

## Material and methods

### Data collection

The TraumaRegister DGU® of the German Society for Trauma Surgery was established in 1993. The aim of this multicenter database is a pseudonymized and standardized documentation of severely injured patients. Data are collected prospectively in four consecutive phases: A) prehospital phase, B) emergency trauma room and subsequent surgical phase, C) intensive care unit, and D) hospital discharge. Documentation includes detailed information on demographics, injury patterns, comorbidities, prehospital and clinical management, course of intensive care, major laboratory findings including transfusion data, and outcome. Inclusion criteria are accident-related admission to the hospital via the emergency trauma room followed by intensive care or intermediate care monitoring, or arrival at the hospital with vital signs and death before admission to the ICU. The infrastructure for documentation, data management, and data analysis is provided by AUC—Academy of Trauma Surgery GmbH, which is affiliated with the German Society of Traumatology (DGU). Scientific leadership is provided by the DGU Section for Emergency, Intensive Care and Serious Injury Care (Section NIS). Via a web-based application, participating hospitals enter their pseudonymized data into a central database. Scientific investigations of the registry data are approved after a review process by the NIS section. Participating hospitals are primarily located in Germany (90%), but an increasing number of hospitals from other countries also contribute data (presently Austria, Belgium, Finland, Luxembourg, Slovenia, Switzerland, the Netherlands, and the United Arab Emirates). Currently, approximately 28,000 cases (base cohort) from nearly 700 hospitals are entered into the database annually. Participation in the TraumaRegister DGU® is voluntary. For the hospitals belonging to the TraumaNetwork DGU® (“*trauma network”),* at least the entry of a basic data set for quality assurance is mandatory. However, about half of all cases are recorded with the more comprehensive standard data set.

### Evaluation of health-related quality of life

**Fig. 1:**
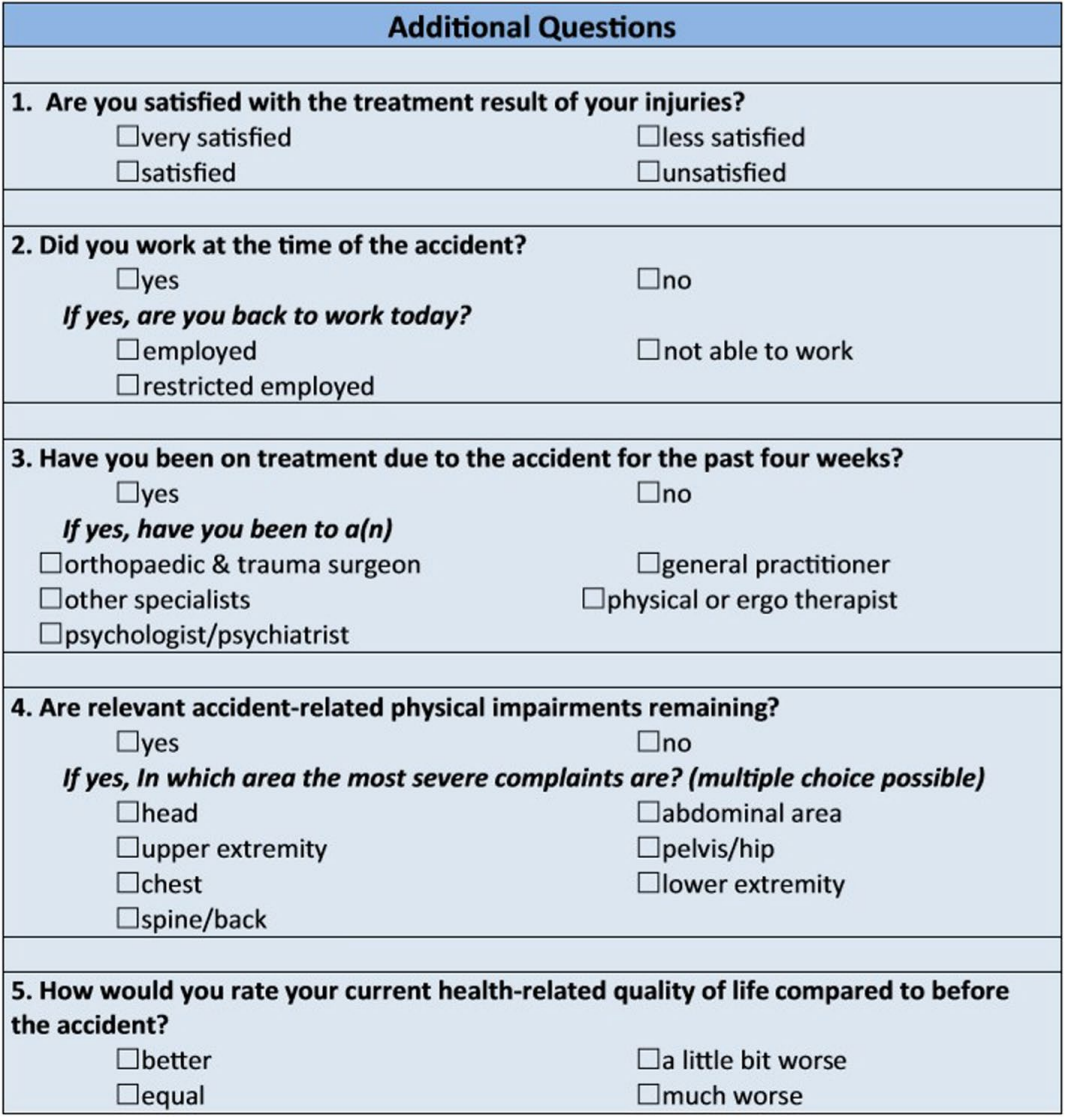
Supplemental questions assessing the treatment satisfaction, ability to work, subjective functional limitations, and health-related quality life 12 months post-injury compared to prior to injury

In 2018, following the already established phases A)—D), an additional phase for the follow-up of severely injured patients, called “E) Outcome”, was developed [20]. The 12-item Short-Form Health Survey (SF-12) was used to measure hrQol of life. Results are presented with two summary scales: the Mental Component Score (MCS-12), and the Physical Component Score (PCS-12). Both summary scales were calculated as a weighted sum according to the manual of the German version of the SF-36 (including SF-12) [21]. Results are presented as mean with standard deviation. Mean values around 50 points represent the average normal population while lower values indicate a lower quality of life. In addition, "E) Outcome" includes five additional elements that assess the treatment satisfaction, the ability to work, trauma-related medical treatment, relevant physical disability, and hrQoL as compared to prior to injury status (Fig. 1).

After positive ethics committee votes (study number University Witten/Herdecke, Duisburg, Köln, Murnau: 52/2017, Düsseldorf: 6198R, Koblenz: 2018–13358, Münster: 2018–215-f-S, Wien: 1688/2017), data collection was started in six German hospitals in 2018. For this purpose, patients who met the inclusion criteria were recruited via the TraumaRegister DGU®. The following inclusion criteria were defined: 1) initial treatment in one of the six participating hospitals; 2) Injury Severity Score (ISS) ≥ 16; 3) age 18–75 years; 4) discharged alive from the study hospital after acute care. Patients who were transferred early (< 48 h after injury) were excluded. Eleven months after injury, the patients were sent an information letter and a consent form for participation by the responsible study hospital. After informed consent, patients were asked to complete the above-mentioned questionnaires in writing. Both questionnaires (SF12 and additional questions) were recorded pseudonymously in the database of the TraumaRegister DGU®. Cases that were not processed or entered by the participating hospital by 18 months after trauma were automatically closed and therefore, not included in the analysis. The data inclusion of the pilot phase covered the period from 01/2017—12/2020.

### Data analysis

For the present analysis, the data were analyzed in aggregated, anonymized form using SPSS (Version 25, IBM Inc., Armonk NY, USA). The descriptive analyses were performed by using number of cases and percentages or means and standard deviation (SD). Differences of categorical characteristics were tested with the chi-square test, and differences in metric variables were tested with the Mann–Whitney U test. A *p* value < 0.05 was considered significant.

## Results

### Study population

For the period 01/2017—12/2020, a total of 1,969 patients (total cohort) met the inclusion criteria in the seven participating clinics. Thus, the total collective was composed of 1,505 (76.4%) male patients and 464 (23.6%) female patients. The age distribution corresponded to the overall cohort of the TraumaRegister DGU® and showed a maximum of 501 (25.4%) patients in the age group 50–59 years. 1,067 (54.2%) of the 1,969 patients had an ISS of 16–24. In 73 (3.7%) patients, the ISS was greater than 50. Table 1 summarizes the baseline characteristics of the cohort.

**Table 1:**
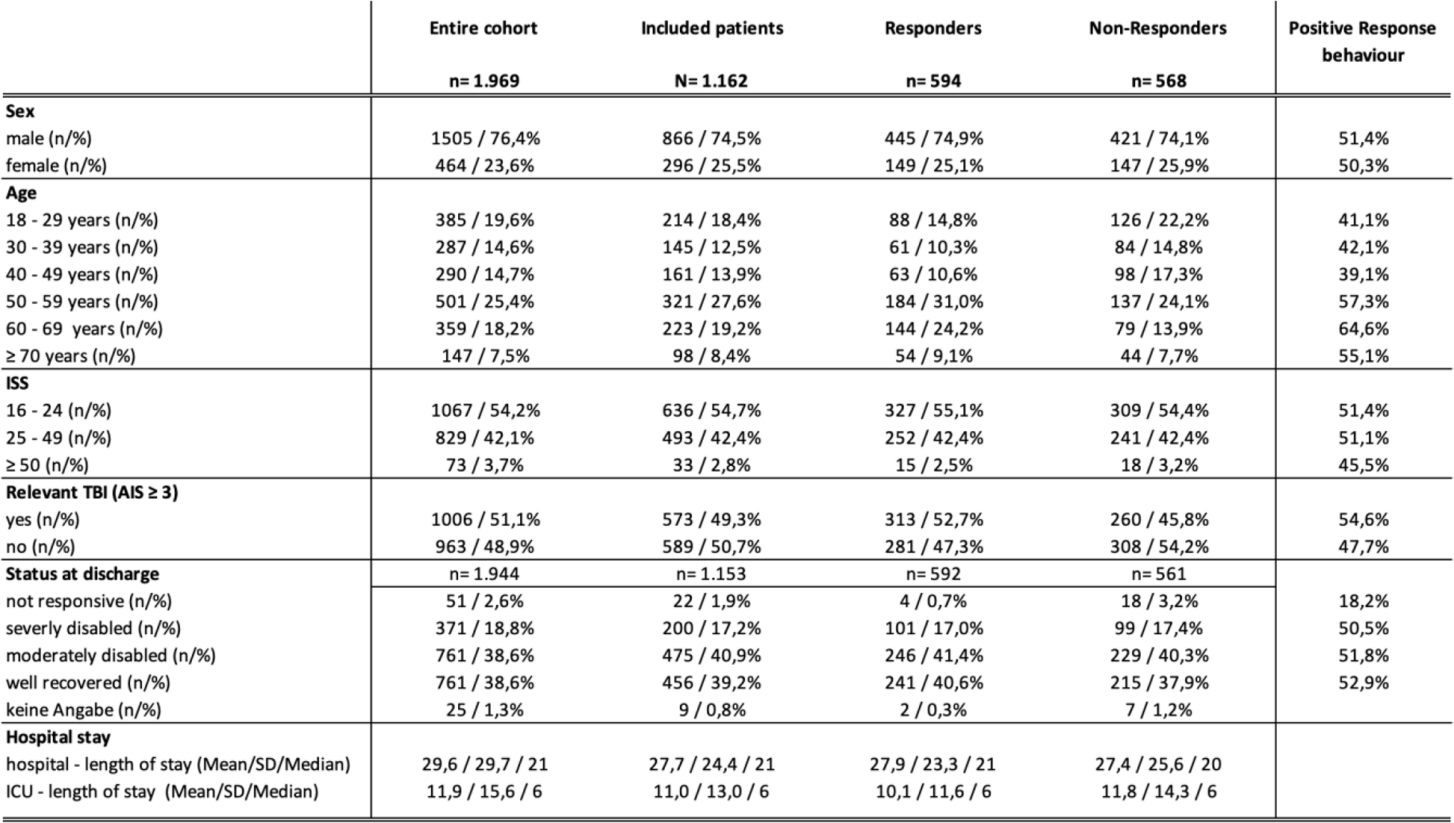
Baseline characteristics of included patients

*Legend*: *ISS* Injury Severity Score, *SD* Standard Deviation, *TBI* traumatic brain injury

**Fig. 2:**
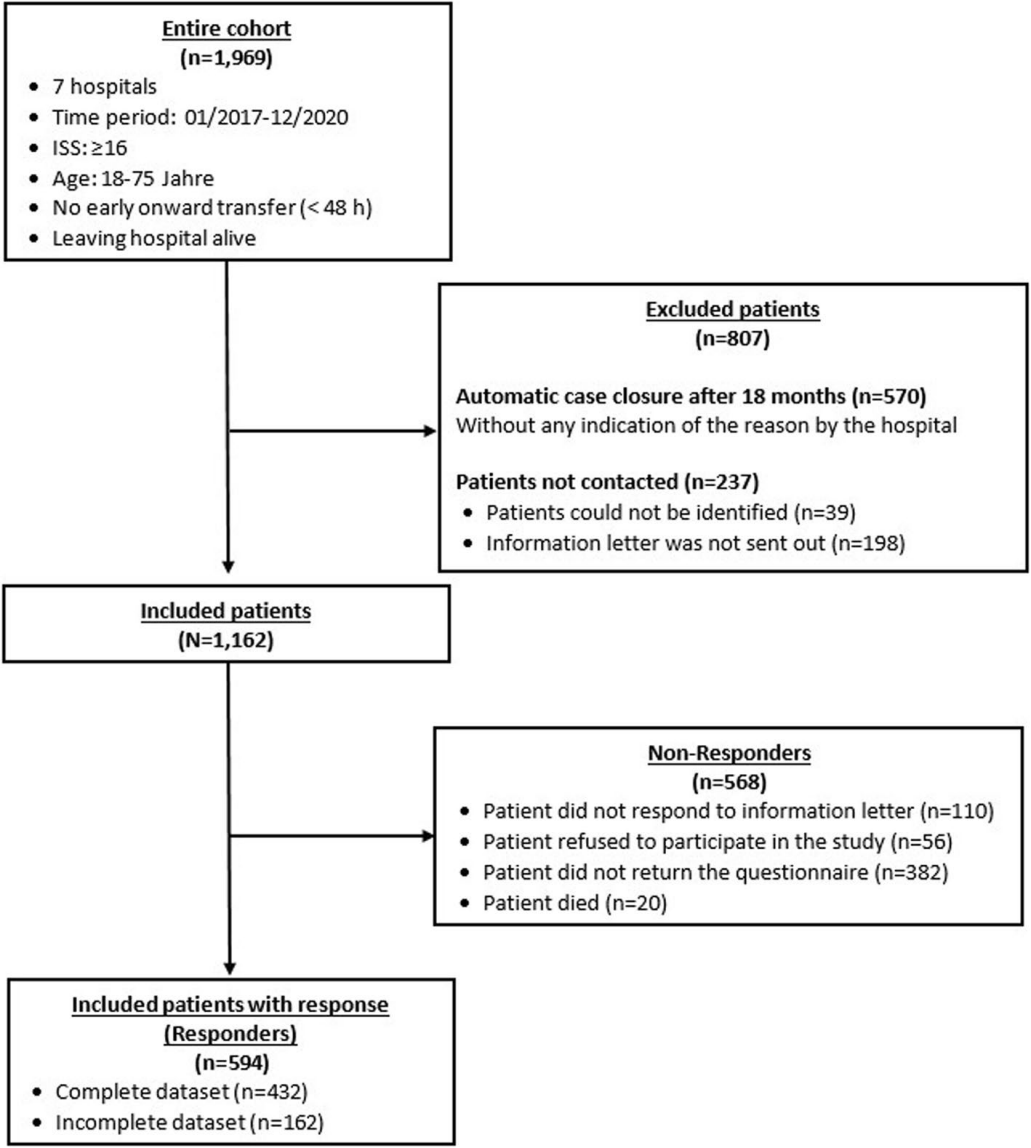
Flowchart with inclusion criteria of the identified population sample and the flow of included and excluded patients

Of the 1,969 patients, 1,162 patients (N) could be contacted by the study clinics and were included in the analysis of the present study. Hence, from the entire cohort 807 patients had to be excluded as they could either not be identified or were not contacted for other reasons. From the 1,162, 866 (74.5%) male and 296 (25.5%) female patients were included. The survey that was conducted in writing and send out via mail, was returned a total of 594 patients. A complete data set was available in 432 cases (72.7%). An overview of the patient flow can be found in Fig. 2.

### Response behavior

In the following, the response behavior, and the number of responders in comparison to the non-responders were examined in relation to various parameters. The response rate was equally distributed among the sex. Of the 866 male seriously injured patients, a total of 428 (49.4%) responded. Among females, the response rate was 49.7% (147 out of 296). In terms of age groups, the distribution of positive response was as follows: 18–29 years: 40.7%; 30–39 years: 40.0%; 40–49 years: 37.9%; 50–59 years: 57.0%; 60–69 years: 60.5%; 70 years and older: 52.0%.

Severely injured patients with an ISS > 50 were less likely to respond (45.5%) than patients with a lower ISS (48.7% for ISS 25–49 and 50.3% for ISS 16–24). Patients who suffered relevant traumatic brain injury (Abbreviated Injury Scale (AIS)-severity head ≥ 3) responded in higher proportions (52.9%) than patients without relevant traumatic brain injury (46.2%). Only 4 of the 22 (18.2%) patients who were unresponsive at discharge responded. Overall, the better the physical status at discharge, the higher was the response behavior. An overview of the response data is summarized in Table 1. Furthermore, the response behavior was related to the injury pattern. Here it can be seen that patients with severe head injuries respond less frequently. Otherwise, a comparable injury pattern can be observed among responders and non-responders (Table 2).

**Table 2:**
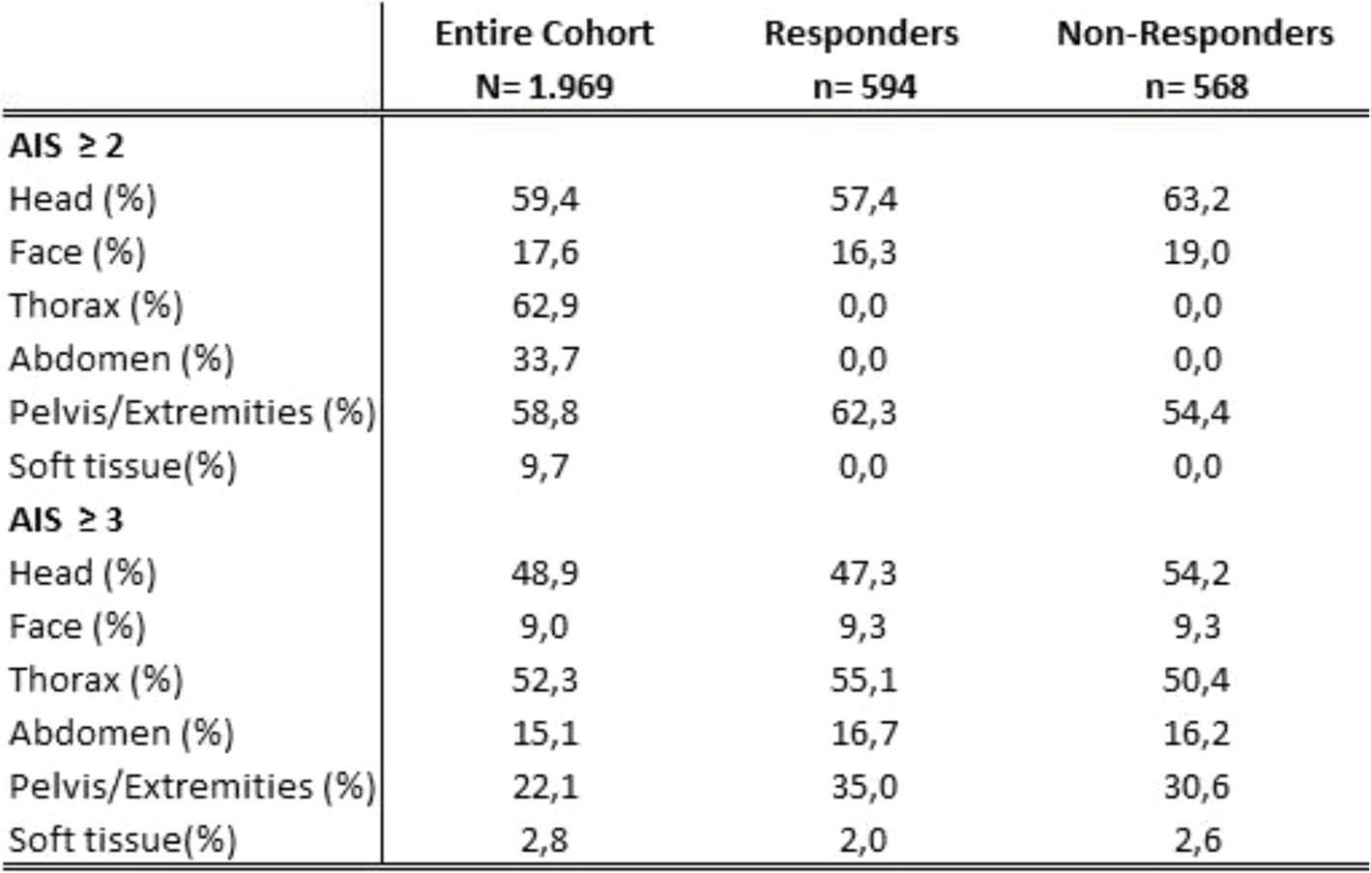
Response behavior of patients related to injury pattern

*Legend*: *AIS* Abbreviated Injury Scale

### Posttraumatic health-related quality of life

No statistically significant differences between sex for the SF-12 physical (PCS), as well as for the SF-12 mental (MCS) sum score (Table 3). However, with regard to age statistical significance is evident with respect to the PCS. The younger the patient at injury, the better the physical sum score, but with no statistically significant difference in the MCS. Furthermore, the physically perceived quality of life decreases significantly in relation to the severity of the injury as measured by the ISS, whereas the mentally perceived quality of life shows no differences in terms of injury severity (Table 3).

**Table 3:**
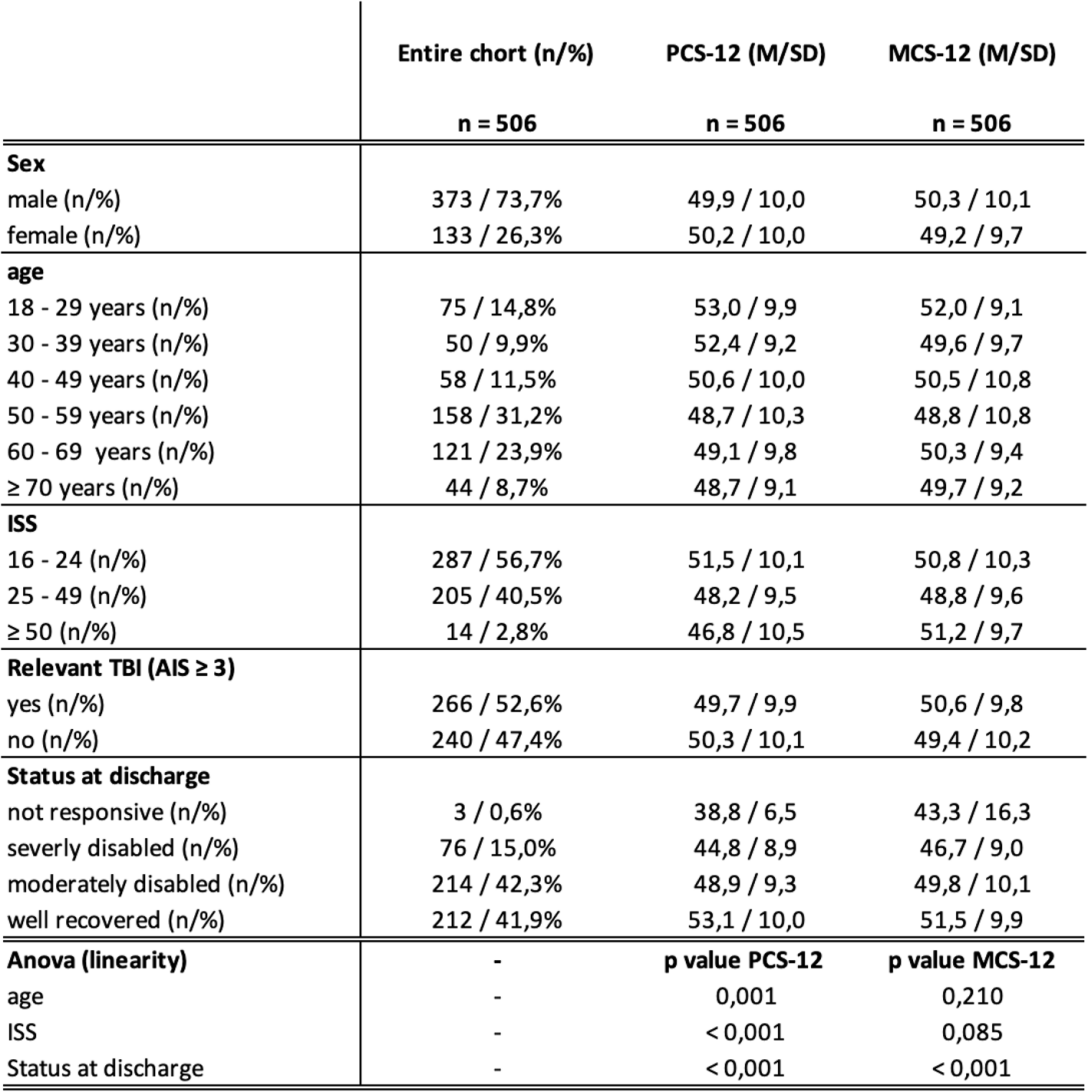
Health-related quality of life measured by SF-12 questionnaire

*Legend: AIS* Abbreviated Injury Scale, *ISS* Injury Severity Score, *PCS-12* Short-Form 12 Physical Component Score, *MCS-12* Short-Form 12 Mental Component Score, *M* mean, *TBI* traumatic brain injury, *SD* standard deviation

**Fig. 3:**
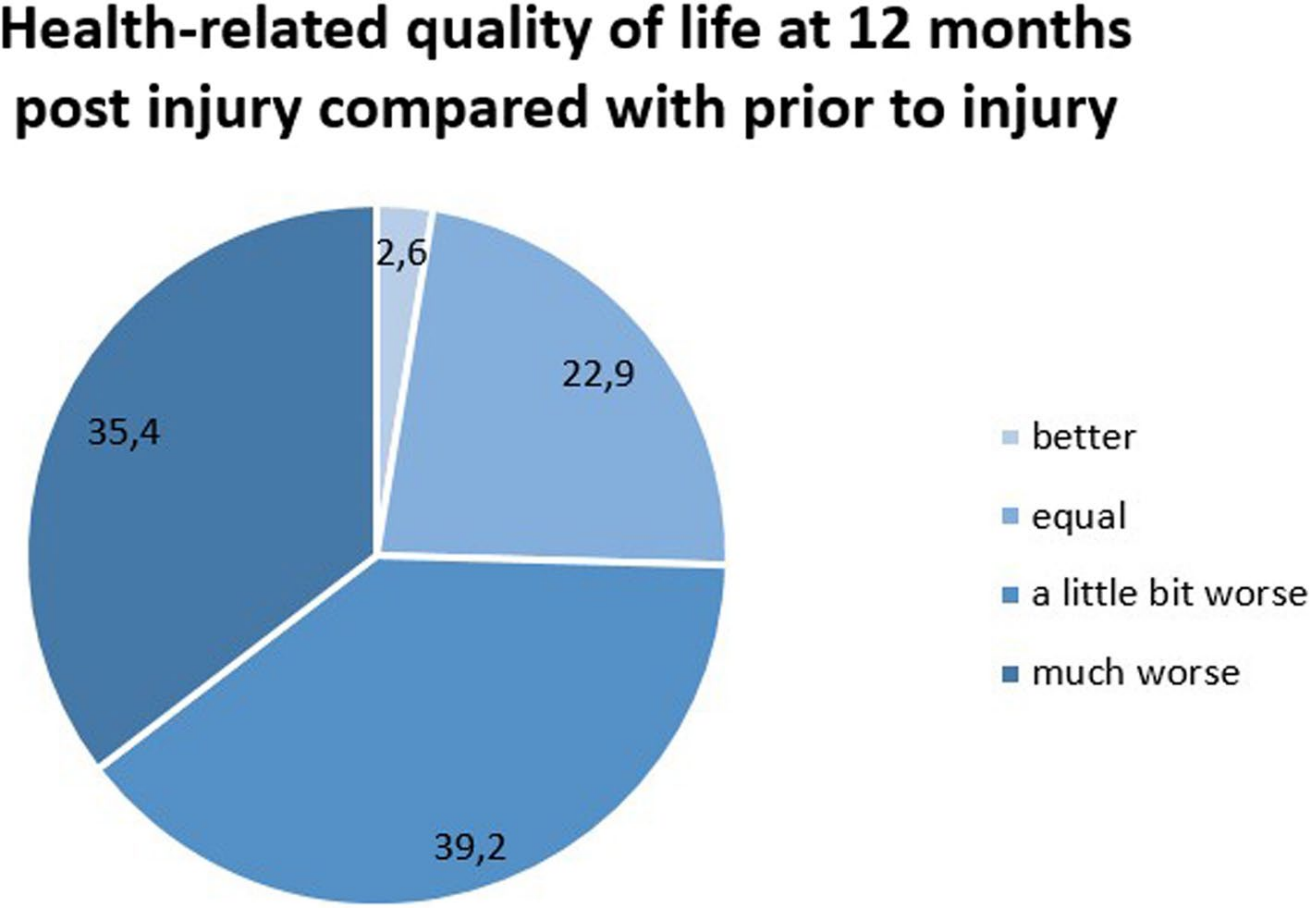
Patient-assessed health-related quality of life at 12 months post injury compared with prior to injury (*n* = 582)

As part of the supplementary questionnaire, patients were asked to compare their current hrQoL with the status prior to injury. 582 of the 594 patients responded to this question. In 2.6%, the hrQoL post-injury was better than prior to injury, and in 22.9% it was reported as broadly the same. A somewhat worse hrQoL was reported by 39.2%, and a much worse hrQoL after the trauma was noted in 35.4% (Fig. 3).

### Employment

**Fig. 4:**
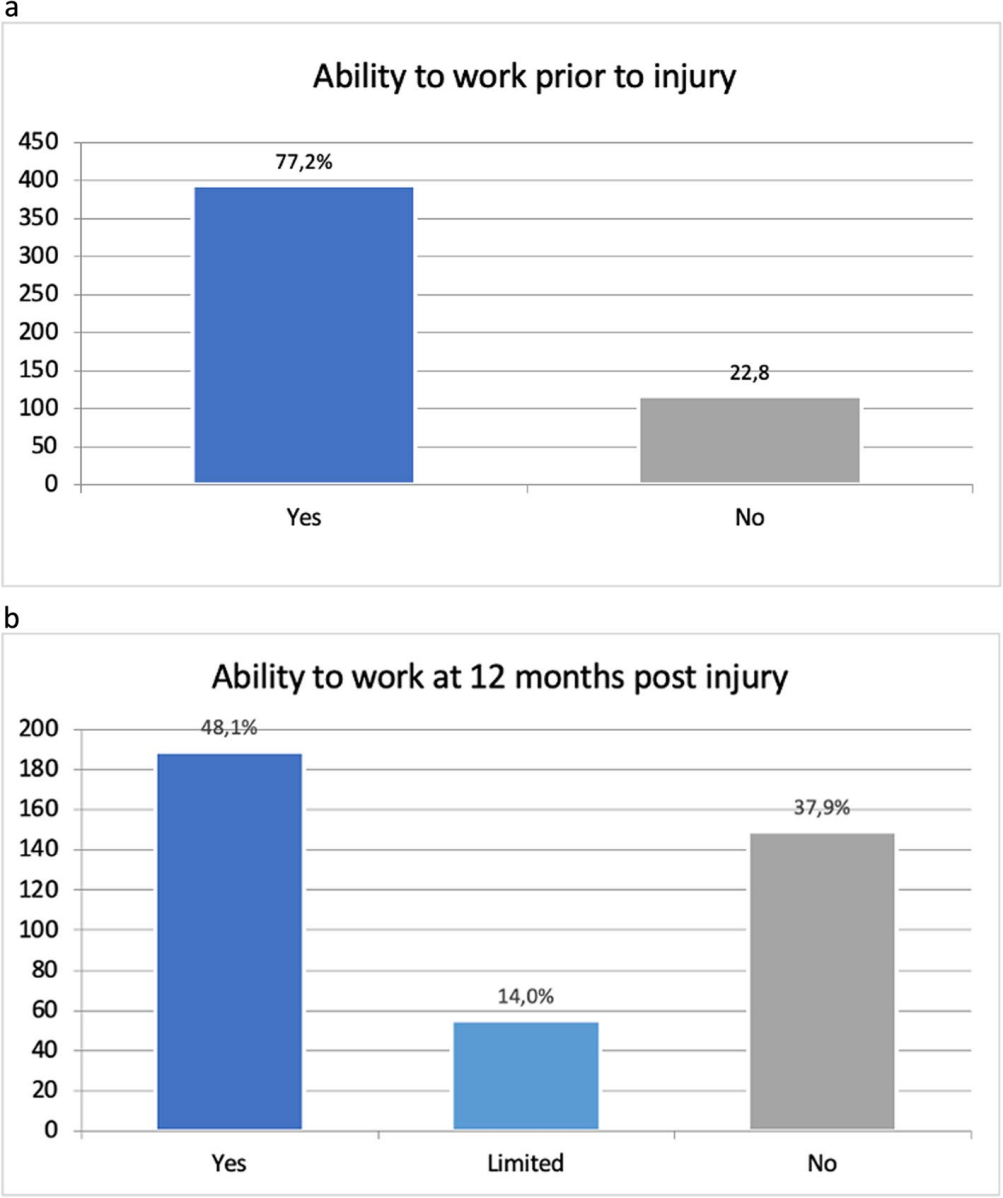
**a** Ability to work prior to injury *n* = 523. **b** Ability to work at 12 months post injury *n* = 429

The ability to work prior to injury and 12 months post-injury was investigated. Of the 594 patients included, 523 provided information regarding their ability to work at the time of the injury. Of these, 407 (77.8%) patients reported that they were able to work prior to injury and 116 (22.8%) patients reported that they were unable to work prior to injury (Fig. 4a).

A total of 429 patients provided information on their ability to work 12 months post-injury. This shows full employment in 194 (45.2%) patients, restricted employment in 58 (13.5%) patients, and an inability to work in 177 (41.3%) patients (Fig. 4b).

### Satisfaction with treatment

**Fig. 5:**
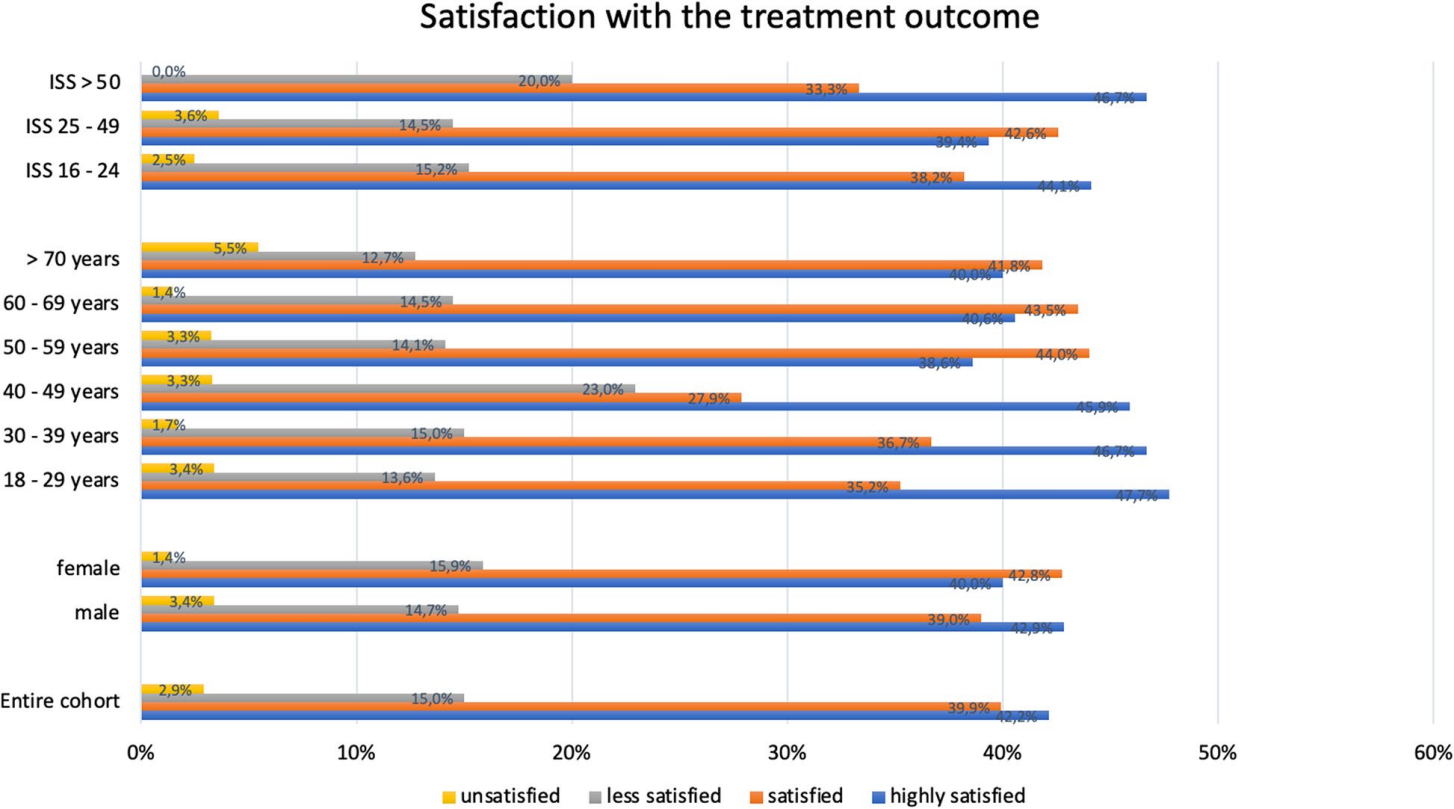
Treatment satisfaction—all included patients and subgroups. Legend: ISS = Injury Severity Score

A total of 586 of the 594 included patients provided information concerning the treatment satisfaction. Most of the severely injured patients were very satisfied (42.2%) or satisfied (39.9%) with the outcome of treatment. Only 15.0% were less satisfied and 2.9% were not at all satisfied with the outcome of treatment of their injuries. Regarding the variable sex, a homogeneous distribution pattern was discovered. Younger patients (< 50 years) were on average more satisfied with the treatment outcome. It should be emphasized that patients with an ISS > 50 (*n = *15), were on average more often very satisfied with the treatment outcome. Figure 5 summarizes the satisfaction with the treatment in relation to the variables mentioned.

## Discussion

The present pilot study demonstrates the successful implementation of the recording of outcome parameters in the existing and well-established TraumaRegister DGU®. Moreover, first findings on hrQoL 12 months after severe injury in a larger cohort were obtained.

The systematic collection of data of severely injured patients in standardized registries has already led to considerable progress worldwide in terms of structural, procedural, and outcome-oriented quality assurance as well as in science. Hitherto, however, virtually all national data collections have been limited to the prehospital and clinical course until the patient is discharged from the respective acute trauma center. Previous outcomes-related post-discharge data collections have been limited either to smaller study populations or to specific injury patterns that are often predefined. This is making it impossible to draw conclusions about how to modify acute care, to improve functional outcomes, and to enhance hrQoL for survivors of severe injury. This gap in care research has already been identified by several research groups [22, 23]. In 2011, the Victorian State Trauma Registry (VSTR) and the “REcovery after Serious Trauma Outcomes, Resource use, and patient Experiences (RESTORE)” project allowed the first registry-based follow-up of severely injured patients to be collected at the state level in Australia [24, 25]. In this study, the available data were limited to a restricted period from July 2011 to June 2012 [24]. The EQ-5D-3L was used to describe hrQoL. In summary, based on their results, the authors were able to postulate that there is a high prevalence of persistent problems even 3 years after polytrauma and thus, the consequences of polytrauma should be considered a chronic condition.

As the first European registry, the TraumaRegister DGU® has succeeded in recording parts of the post-discharge progression of severely injured patients with the “ E) Outcome” [20]. Advantages of the registry-based data collection are the pseudonymized, standardized, and time-saving data collection. Furthermore, after completion of the pilot phase, an expansion to all trauma centers participating in the TraumaRegister DGU® and thus, a temporally unlimited follow-up of all patients recorded in the TraumaRegister DGU® would be possible.

During the pilot phase, data were collected by mail. The advantages of this type of survey are the data privacy compliant procedure and the cost savings, but mostly at the expense of the response rate. The average response rate in this study was 32%. A closer look at the response behavior shows a lower response rate among the younger patient groups (< 50 years). However, a non-response bias (selection bias) cannot be ruled as patients may not have responded because they were too ill, unable to complete the questionnaire, for example, as a result of arm/hand or head injuries, or as a result of depression and/or posttraumatic stress disorder. This could have affected the results as responding patients could have recovered better or faster than non-responders which might have led too overly positive results. The comparison with the literature shows that in polytrauma research the response rates are on average 50–58% also because a great part of patients died within the first two to five years post-injury [26, 27].

An e-mail- or app-based survey could address here especially the younger patient groups, be more cost-saving in the long term, and more flexibly applied in various languages [28]. By using different methods (postal, telephone, web-based), the response rate could certainly be increased. As expected, the response rate of patients with severe head injuries (AIS ≥ 2) was correspondingly lower.

In order to increase the response rate in the patient population examined, it would have been possible to use a proxy to answer the questionnaire. However, the use of proxies to answer the questions after polytrauma is rarely reported [14]. Injuries such as traumatic brain injuries may even require the use of a proxy. Proxy and patient questionnaires are analyzed equally but health status may differ in their responses [29]. In this context, Gabbe et al. showed that the differences between patients and proxy questioning show a random variability rather than systematic bias. Therefore, proxy questionnaires could suffer from bias when assessing the individual patient recovery, but they are unlikely to bias the group comparisons [30]. Hence, the utilization of proxies could enable the collection of outcome data after polytrauma, despite a risk of bias [31].

The examination of post-injury hrQoL is one of the central elements of the outcome questionnaire. The present results show that the younger the patient is at the time of the injury, the higher his physical sum score one year after polytrauma. In severe polytrauma with a high ISS, the physical perceived quality of life is statistically significantly impaired, where, on the other hand, the mental perceived quality of life shows no differences with respect to injury severity. Very severely injured patients are still physically limited 1 year after the injury, but with a well-compensated psyche. This also coincides with the satisfaction of the treatment outcome (Table 3). Patients with high injury severity (ISS > 50) are more satisfied than the average (Fig. 5). A similar conclusion was reached by the working group around Havermanns and colleagues [29]. Younger age and extremity injury were detected as short-term prognostic for a lower health status after a severe trauma. Whereas unemployment prior injury and comorbidities were shown to be long-term prognostic factors for a reduction in hrQoL.

A limitation of this pilot study is the use of a single generic instrument to measure hrQol, the SF-12. Ritschel et al. were able to show in their systematic review that there is a large variation in the assessment of patient reported outcomes after polytrauma, but the generic instruments SF-12 and SF-36 are the most widely used questionnaires in polytrauma research [14]. The introduction and further development of questionnaires such as the POLO chart as a specific polytrauma hrQoL tool based on patient- and expert- interviews is planned for future studies [32].

## Conclusion

In light of the present results, it must be postulated that a structured assessment of the post-injury hrQoL of severely injured patients must considered or should include various pre- and post-injury factors. The perspective of some authors to evaluate long-term problems after severe injury—be it physical or psychological—as a chronic condition must be taken into account, as well as the detection of structural and procedural weaknesses, which must be improved for the benefit of the affected patients.

## Data Availability

The data that support the findings of this study are available from AUC GmbH but restrictions apply to the availability of these data, which were used under license for the current study, and so are not publicly available. Data are however available from the authors upon reasonable request and with permission of AUC GmbH.
